# Heterogeneity of weight gain after initiation of Elexacaftor/Tezacaftor/Ivacaftor in people with cystic fibrosis

**DOI:** 10.1186/s12931-023-02451-0

**Published:** 2023-06-17

**Authors:** Andrea Gramegna, Fabio Majo, Gianfranco Alicandro, Gloria Leonardi, Luca Cristiani, Francesco Amati, Martina Contarini, Stefano Aliberti, Alessandro Giovanni Fiocchi, Francesco Blasi

**Affiliations:** 1 Department of Pathophysiology and Transplantation, University of Milan, Milan, Italy; 2 Internal Medicine Department, Respiratory Unit and Cystic Fibrosis Adult Center, Fondazione IRCCS Ca’ Granda Ospedale Maggiore Policlinico, Milan, 20122 Italy; 3 Cystic Fibrosis Unit, Bambino Gesù Children’s Hospital, IRCCS, Rome, Italy; 4 Department of Biomedical Sciences, Humanitas University, Pieve Emanuele, MI Italy; 5 Respiratory Unit, IRCCS Humanitas Research Hospital, Rozzano, MI Italy

**Keywords:** Weight gain, Response to treatment, Personalized medicine, CFTR modulators, Elexacaftor, Overweight, Adults

## Abstract

**Background:**

The introduction of the novel therapy, Elexacaftor/Tezacaftor/Ivacaftor (ETI) has been effective in improving weight gain in both clinical trials and real-world studies. However, the magnitude of this effect appears to be heterogeneous across patient subgroups. This study aims to identify potential determinants of heterogeneity in weight gain following 6-month ETI therapy.

**Methods:**

We conducted a multicenter, prospective cohort study enrolling 92 adults with CF at two major CF centers in Italy with follow-up visit at one month and six months from ETI initiation. The treatment’s effect on weight changes was evaluated using mixed effect regression models that included subject-specific random intercepts and fixed effects for potential predictors of treatment response, time and a predictor-by-time interaction term.

**Results:**

The mean weight gain at six months from the start of treatment was 4.6 kg (95% CI: 2.3–6.9) for the 10 patients with underweight, 3.2 kg (95% CI: 2.3-4.0) for the 72 patients with normal weight, and 0.7 kg (95% CI: -1.6-3.0) for the 10 patients with overweight. After six months of ETI treatment, 8 (80%) of the patients with underweight transitioned to the normal weight category, while 11 (15.3%) of the normal-weight patients became overweight. The major determinants of heterogeneity in weight gain were the baseline BMI and the presence of at least one CFTR residual function mutation, explaining 13% and 8% of the variability, respectively.

**Conclusions:**

Our results indicate that ETI is highly effective in improving weight gain in underweight subjects with CF. However, our data also suggests the need for close monitoring of excess weight gain to prevent potential cardiometabolic complications.

**Supplementary Information:**

The online version contains supplementary material available at 10.1186/s12931-023-02451-0.

## Highlights

We reported heterogeneity in weight gain after 6 months of treatment with ETI.

Baseline BMI and CFTR genotype predict heterogeneity in treatment response.

People with underweight received the greatest benefit from ETI treatment.

An amount of 15% of normal-weight subjects became overweight.

**Supplementary Information:**

The online version contains supplementary material available at 10.1186/s12931-023-02451-0.

## Background

In recent years, the landscape of nutritional outcomes for people with cystic fibrosis (CF) has undergone significant changes [1]. Traditionally, body mass index (BMI) has been considered the main indicator for nutritional status in people with CF. Low BMI levels have been linked to an accelerated decline in forced expiratory volume in one second (FEV1), a higher rate of pulmonary exacerbations and increased mortality [2]. As a result, international guidelines recommend that adult females achieve a BMI target of ≥ 22 kg/m^2^, and adult males a target of ≥ 23 kg/m^2^ [1].

Advances in CF treatment, including aggressive nutritional interventions, have led to increased life expectancy. However, overweight and obesity have emerged as a growing concern, especially in adults and in high-income countries [3, 4]. The recent development of highly effective CFTR modulator therapy, such as Elexacaftor/Tezacaftor/Ivacaftor (ETI), is thought to have contributed to this trend. Clinical trials and real-world studies have shown that ETI is associated with improved BMI. In the registrational trials, ETI led to a significant increase in BMI after 24 weeks of treatment (1.04 kg/m^2^ versus placebo in Phe508del heterozygous and 0.60 kg/m^2^ versus Tezacaftor/Ivacaftor in Phe508del/Phe508del, respectively) [5, 6]. In a series of real-world observational studies, ETI was found effective in increasing BMI across different patient groups, including those with advanced lung disease [7, 8]. However, the response to ETI varied significantly, and factors potentially related to BMI response have not yet been studied.

The aim of this Italian multicenter study was to investigate predictive factors for weight gain following ETI treatment and to evaluate the risk of excessive weight gain.

## Methods

### Study design and data collection

This was an observational, prospective, longitudinal multicenter study. Consecutive adults (≥ 18 years) with CF who started treatment with ETI between 2021, January and 2022, June enrolled at two major CF Centers in Italy (IRCCS Ca’ Granda Ospedale Maggiore Policlinico, Milan; Ospedale Pediatrico Bambin Gesù, Rome). Data were prospectively collected by two qualified investigators at baseline and after 1 and 6 months from ETI initiation. The investigators were provided with a protocol including the following study definitions. The study protocol was approved by local institutional review boards (594_2016bis) and all participants provided written informed consent to take part in the study. This project had no funding and relied upon voluntary participation.

### Study definitions

CF was defined following the standard diagnostic criteria [9]. Minimal function mutations and residual function mutation were defined according to the list of mutations included in the registration studies of ETI and Tezacaftor/Ivacaftor, respectively [5, 10]. BMI categories were defined according to the WHO criteria as follows: underweight (< 18.5 kg/m^2^), normal weight (BMI: 18.5–24.9 kg/m^2^) and overweight (BMI ≥ 25.0 kg/m^2^) [11]. Chronic P. *aeruginosa* infection was defined by the isolation of P. *aeruginosa* in sputum culture in more than 50% of samples over the last 12 months with a minimum of three samples. Referring to comorbidities, pancreatic insufficiency was defined in patients treated with pancreatic enzymes, while CF-related diabetes (CFRD) was diagnosed according to oral glucose tolerance test. Treatment response was evaluated as changes in weight and BMI from pre-treatment values.

### Statistical analysis

We examined several factors that could potentially affect treatment response including sex, age group (< 40 vs. ≥ 40 years), carrying a residual function mutation, pancreatic insufficiency, P. *aeruginosa* colonization, severe impairment of respiratory function (as defined by percent of predicted FEV1, ppFEV1 < 40), BMI category and CFRD. To estimate the effect of each factor on treatment response we used mixed effects regression models that included subject-specific random intercepts and fixed effects for the potential predictors of treatment response, time and a predictor-by-time interaction term. The likelihood ratio test was used to determine the statistical significance of the interaction term by comparing two nested models - one with the interaction term and one without. A significance test result indicates that the treatment response varied between levels of the predictor. We computed the marginal R^2^ of the model with the interaction term as an estimate of the total variability explained by each potential predictor [12]. The Pearson’s correlation coefficient was used to evaluate the correlation between 6-month changes in BMI and ppFEV1.

We reported point estimates with two-sided 95% confidence intervals (CI), and all statistical tests were conducted with a significance level of α = 0.05.

## Results

### Patient characteristics

Ninety-two consecutive patients (60 males) were included in the study, with a median age of 38 years (range: 25–57). The patients’ characteristics are reported in Table 1. Approximately 70% of the patients were pancreatic insufficient, 64% had chronic respiratory infections caused by P. *aeruginosa*, 25 patients (27.8%) had a severely impaired respiratory function, 10 patients (10.9%) were underweight and 10 were overweight (10.9%).

**Table 1:** Baseline characteristics

Characteristic	N = 92^1^
Sex	
Males	60 (65.2%)
Females	32 (34.8%)
Age (years)	
Median (IQR)	38 (33; 43)
Age category	
< 40 years	53 (57.6%)
≥ 40 years	39 (42.4%)
Residual function mutation	12 (13.0%)
Pancreatic insufficiency	64 (69.6%)
Chronic P. *aeruginosa* infection	59 (64.1%)
ppFEV1	
Median (IQR)	50.5 (39.0; 70.8)
Unknown	2
ppFEV1 < 40	25 (27.8%)
Unknown	2
CFRD	23 (25.0%)
Previous CFTR modulator therapy	
None	69 (75.0%)
Lumacaftor/Ivacaftor	21 (22.8%)
Tezacaftor/Ivacaftor	1 (1.1%)
Ivacaftor	1 (1.1%)
Height (cm)	
Median (IQR)	170 (163; 175)
Weight (kg)	
Median (IQR)	60.0 (55.7; 70.0)
BMI (kg/m^2^)	
Median (IQR)	21.8 (19.1; 23.5)
BMI category	
Underweight	10 (10.9%)
Normal weight	72 (78.3%)
Overweight	10 (10.9%)

^1^n (%), unless otherwise specified

BMI: body mass index. CFRD: cystic fibrosis-related diabetes. CFTR: cystic-fibrosis transmembrane conductance regulator. IQR: Interquartile range. ppFEV1: percent predicted forced expiratory volume in one second.

### Treatment response

**Fig. 1:**
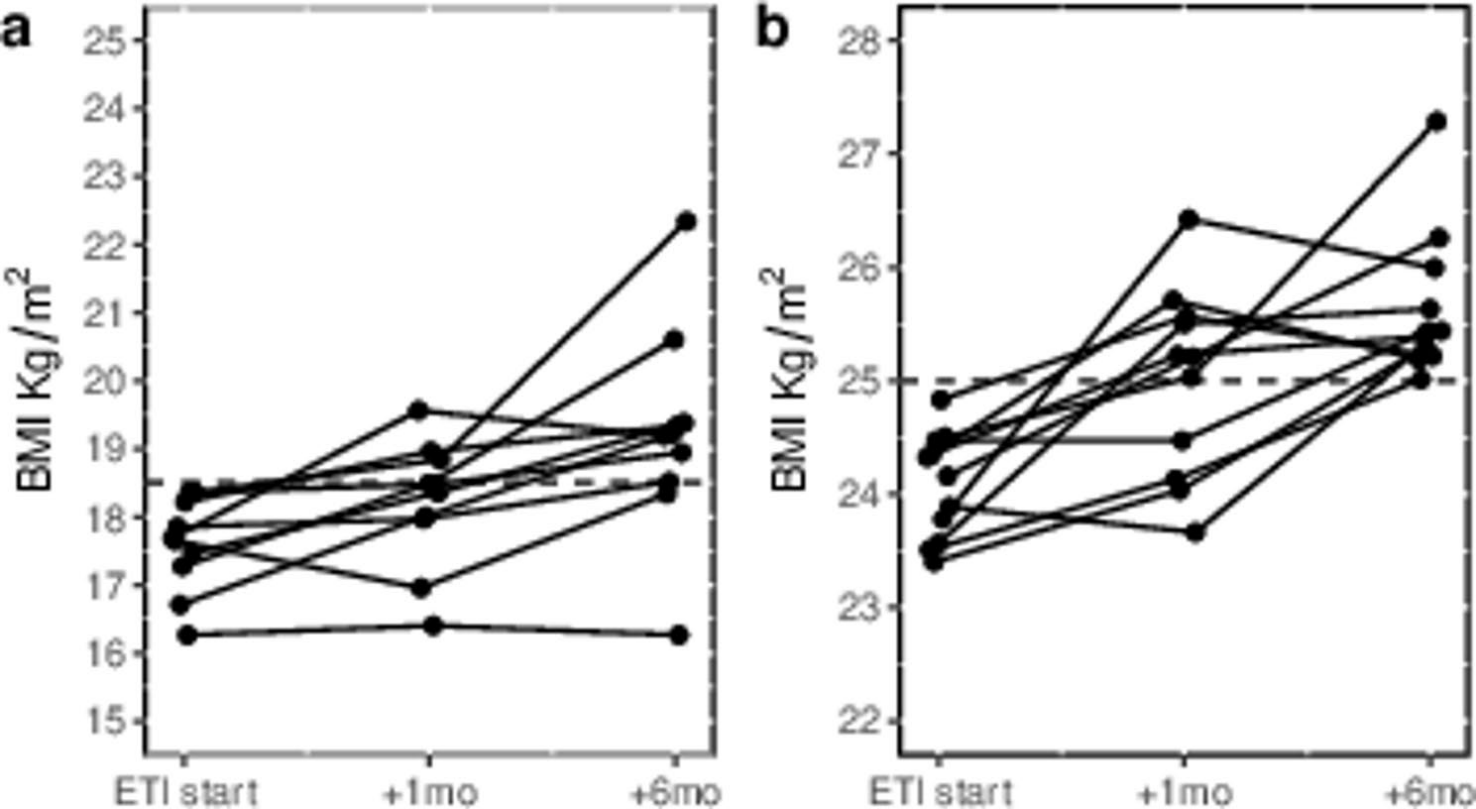
Box plots of changes in body weight and body mass index following Elexacaftor/Tezacaftor/Ivacaftor therapy in patients with cystic fibrosis showing heterogeneity in treatment response

After one month of treatment, body weight increased on average by 1.6 kg (95% CI: 1.0-2.3) and body mass index (BMI) by 0.57 kg/m² (95% CI: 0.34–0.80). At six months from the start of ETI, body weight increased by 3.1 kg (95% CI: 2.4–3.7) and BMI by 1.07 kg/m² (95% CI: of 0.84–1.30). However, the response to treatment varied significantly with changes in body weight ranging from a decrease of 4 kg to an increase of 13.2 kg and changes in BMI ranging from a decrease of 1.2 kg/m² to an increase of 4.0 kg/m² after six months (Fig. 1). Thirteen patients (14.1%) did not experience any increase in body weight during the treatment period. These patients had a lower rate of previous prescription of other CFTR modulators (46.2% vs. 79.7%) and a higher baseline BMI value (23.4 vs. 20.9 kg/m^2^) (Online supplement Table S1).

**Fig. 2:**
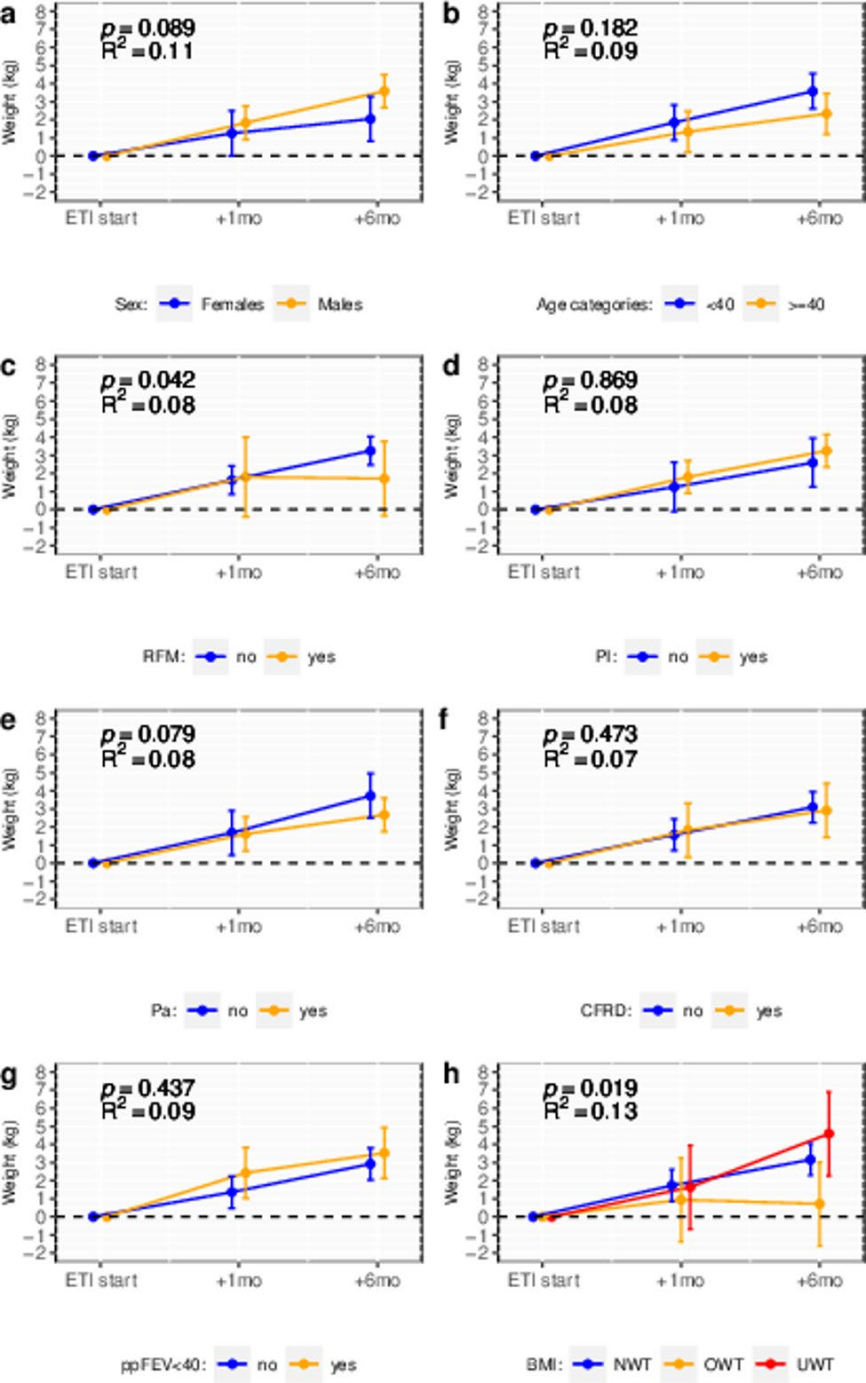
Mean changes in body weight following Elexacaftor/Tezacaftor/Ivacaftor therapy in patients with cystic fibrosis, according to sex (panel **a**), age category (panel **b**), residual function mutation (panel **c**), pancreatic insufficiency (panel **d**), chronic respiratory infection by P. *aeruginosa* (panel **e**), cystic fibrosis related diabetes (panel **f**), respiratory function (panel **g**) and body mass index (panel **h**). Mean changes were estimated using mixed effects regression models including subject-specific random intercepts and fixed effects for the predictor variable, time and a predictor-by-time interaction term. Bars are 95% confidence intervals. *P*-values indicate the statistical significance of the interaction term: i.e. differences in treatment response across levels of the predictor. R^2^ indicates the proportion of the variance in weight changes explained by the fixed effect terms BMI: body mass index. CFRD: cystic-fibrosis related diabetes. ETI: Elexacaftor/Tezacaftor/Ivacaftor. Pa: *Pseudomonas aeruginosa*. PI: pancreatic insufficiency. ppFEV1: percent of predicted forced expiratory volume in one second. RFM: Residual function mutation

**Fig. 3:**
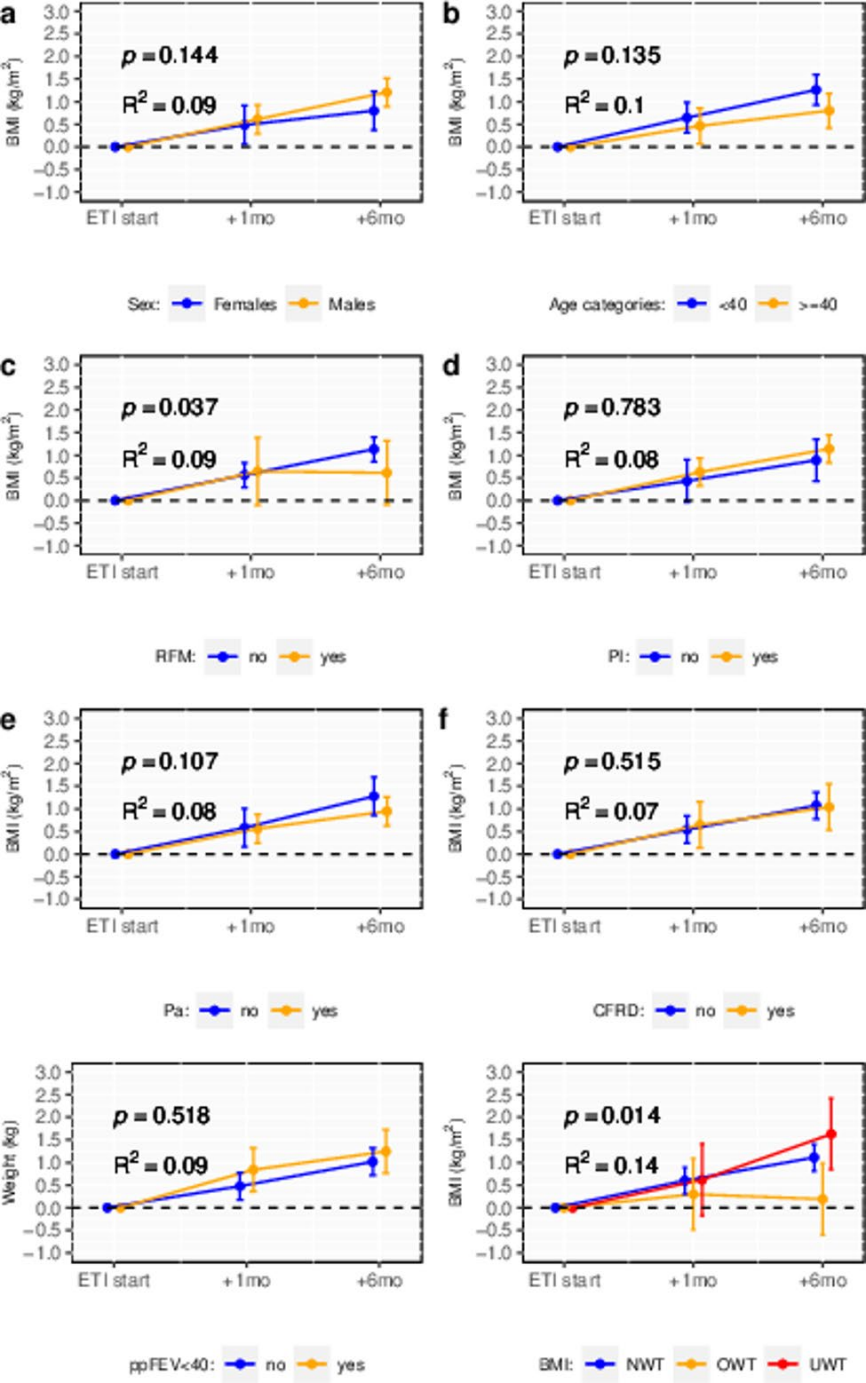
Mean changes in body mass following Elexacaftor/Tezacaftor/Ivacaftor therapy in patients with cystic fibrosis, according to sex (panel **a**), age category (panel **b**), residual function mutation (panel **c**), pancreatic insufficiency (panel **d**), chronic respiratory infection by P. *aeruginosa* (panel **e**), cystic fibrosis related diabetes (panel **f**), respiratory function (panel **g**) and body mass index (panel **h**). Mean changes were estimated using mixed effects regression models including subject-specific random intercepts and fixed effects for the predictor variable, time and a predictor-by-time interaction term. *P*-values indicate the statistical significance of the interaction term: i.e. differences in treatment response across levels of the predictor. R^2^ indicates the proportion of the variance in BMI changes explained by the fixed effect terms BMI: body mass index. CFRD: cystic-fibrosis related diabetes. ETI: Elexacaftor/Tezacaftor/Ivacaftor. Pa: *Pseudomonas aeruginosa*. PI: pancreatic insufficiency. ppFEV1: percent of predicted forced expiratory volume in one second. RFM: Residual function mutation

In Fig. 2, the estimated weight changes are displayed across various levels of the selected potential predictors, while Fig. 3 illustrates the estimates related to changes in BMI.

Higher BMI was associated with lower treatment response in terms of both weight gain and BMI change. Six months after treatment initiation, patients with underweight gained on average 4.6 kg in body weight (95% CI: 2.3–6.9), patients with normal weight gained 3.2 kg (95% CI: 2.3-4.0), and patients with overweight gained 0.7 kg (95% CI: -1.6-3.0). The corresponding figures for BMI were 1.6 kg/m^2^ (0.8–2.4) for patients with underweight, 1.1 kg/m^2^ (0.8–1.4) for patients with normal weight, and 0.2 kg/m^2^ (-0.6-1.0) for patients with overweight. As indicated by the marginal R-squared of the model including time, baseline BMI category and BMI-by-time as fixed effects, BMI at baseline explained 13% and 14% of the variability in weight changes and BMI, respectively.

After six months of ETI treatment, patients carrying a CFTR residual function mutation experienced a lower weight gain (mean change: 1.7 kg, 95% CI: -0.3-3.8) compared to those without the mutation, who gained on average 3.2 kg (95% CI: 2.5-4.0). The corresponding changes in BMI were: +0.6 kg/m^2^ (95% CI: -0.1-1.3) in patients with a residual function mutation and + 1.1 kg/m^2^ (95% CI: 0.9–1.4) in those without the mutation. The variance in treatment response explained by this factor was around 8–9% (Fig. 2**and** Fig. 3).

**Fig. 4:**
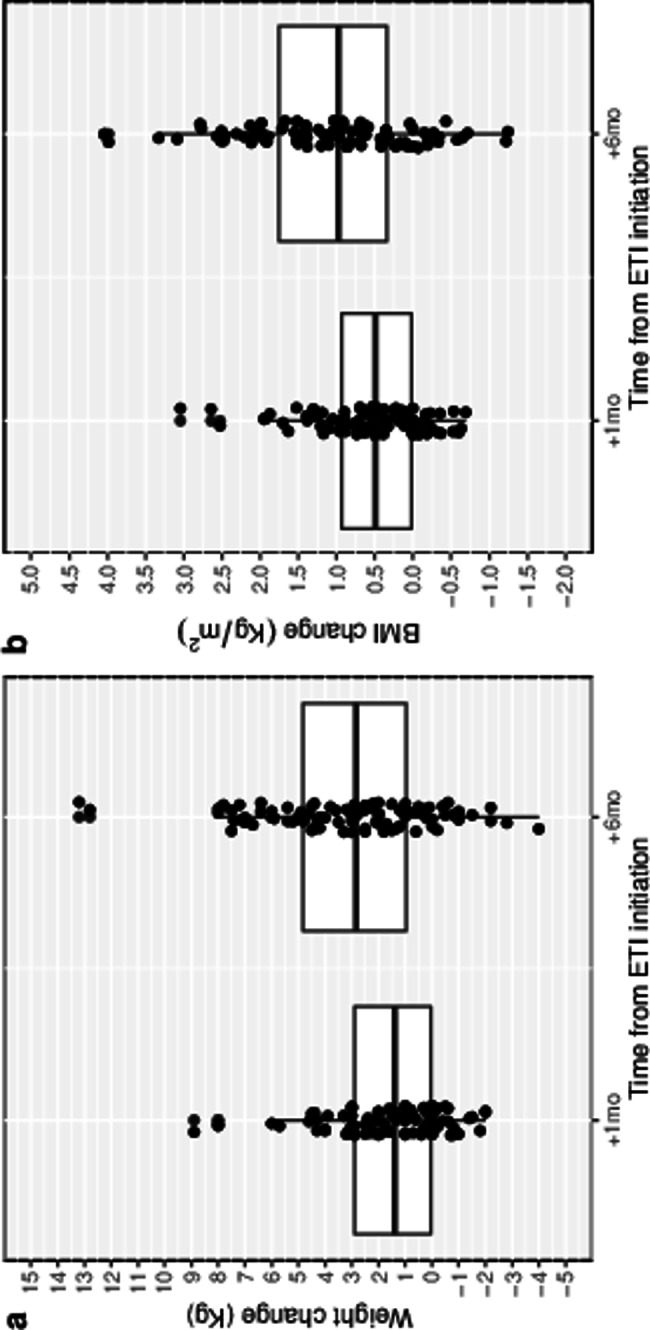
Individual changes in body mass index following Elexacaftor/Tezacaftor/Ivacaftor therapy in patients with cystic fibrosis who were underweight at baseline visit (panel **a**) and among patients who became overweight during the study period (panel **b**)

In addition, after six months of ETI treatment, 8 out of the 10 patients with underweight (80%) at ETI initiation transitioned to the normal weight category, while 11 out of the 72 patients (15.3%) in the normal weight category became overweight (Fig. 4).

The increase in BMI was associated with an improvement in respiratory function, as shown by a mean change of + 14.5 (95% CI: 12.2–16.8) in ppFEV1 after 6 months. This improvement was observed in all BMI categories, with patients with underweight experiencing the most significant improvement (+ 24.5%, 95% CI: 9.3–39.6). However, changes in ppFEV1 showed only a moderate correlation with changes in BMI (r = 0.29, 95% CI: 0.09–0.47).

## Discussion

This study found that in people with CF: (1) weight gain after six months of ETI treatment depends on pre-treatment BMI values and the presence of CFTR residual function mutations; (2) ETI is highly effective in improving weight gain in subjects with underweight; (3) a significant proportion of the treated population experienced excessive weight gain; (4) weight gain is only moderately correlated with improvement in respiratory function.

Since the approval of the drug, clinical research on ETI and its impact on multisystemic involvement of CF has generated a growing body of evidence from real-world reports, including data on changes in BMI and other metabolic parameters [7, 8, 13, 14]. In particular, this aspect has garnered renewed interest due to the recent thought that nutritional management in CF warrants a deep reconsideration [15].

Our study highlights the heterogeneity of weight gain among CF adults following six months of treatment with ETI. We found that baseline BMI is the main determinant of heterogeneity in treatment response, with greater improvements observed in the population with lower BMI at ETI initiation. The metabolic effect of ETI through the restoration of CFTR is therefore exerted with different degrees in people with CF, with the highest responses seen in patients with underweight and malnutrition secondary to CF. Consistent with these results, while post-treatment BMI changed significantly in both the underweight and normal-weight groups, there was no evidence of weight gain among patients with overweight.

This observation raises important considerations. Firstly, the fact that patients with overweight are less susceptible to weight gain is reassuring that ETI may not further worsen the cardio-metabolic risk profile in these patients. However, approximately 15% of patients with normal weight in our cohort became overweight after receiving ETI for six months. This finding along with emerging data on the increasing prevalence of overweight in CF highlights the need for closer attention to excess weight gain in patients treated with ETI [3, 4]. Whether long-term treatment with ETI can increase the incidence of overweight and obesity among people with CF is a topic of current debate [4, 7]. The lack of significant weight gain did not seem to affect the clinically relevant improvement in pulmonary function observed in patients with overweight, indicating that the pathophysiological pathway targeted by ETI for respiratory function and nutritional status may be different.

Distinct response patterns were noticed among patients who had at least one CFTR residual function mutation compared to those with more severe CFTR genotypes. However, there is a shortage of data on this matter, as the phase-3 clinical trial by Barry et al. examining the role of ETI for Phe508del and CFTR genotypes with residual function mutations did not include any data on BMI change [16]. Thus, larger multicenter retrospective studies or studies from national and international registries are needed to better clarify the effects of ETI on weight gain in patients with these CFTR genotypes.

The mechanisms underlying heterogeneity in weight gain following ETI has not been fully understood, and are likely to be multifactorial, including decreased energy expenditure at rest, decreased gut inflammation and improved fat malabsorption [14].

To our knowledge, this is the first study investigating the heterogeneity of drug effects on BMI and which factors are the main determinants. Our unique approach could provide valuable insights for clinical research to investigate the impact of ETI in real-world scenarios. Our findings indicate that baseline BMI and CFTR genotype only account for a modest proportion (8 to 14%) of the variability in treatment response, suggesting that other factors, such as CFTR modifier genes, i.e. variants outside the CFTR locus, may also play a role [17].

The study has several limitations, including a limited number of participants and possible geographical differences that may reduce the generalizability of our findings. Additionally, some factors evaluated in this study did not reach statistical significance to be considered potential determinants of heterogeneity in weight changes after ETI. However, due to the small sample size in some categories, we cannot rule out the possibility that a larger study may yield different results.

Last, we did not evaluate changes in body composition following ETI therapy, which could provide valuable insights into the metabolic mechanisms underlying the effects of ETI on weight gain.

## Conclusions

Baseline BMI is the major determinant of heterogeneity in weight gain in patients with CF treated with ETI, with patients affected by underweight receiving the greatest benefit from this treatment. Our results, along with evidence of increasing prevalence of overweight and its metabolic complications, such as hyperlipidaemia and hypertension [7, 18] suggest a close monitoring of excess weight in patients undergoing ETI therapy.

### Electronic supplementary material

Below is the link to the electronic supplementary material.

Supplementary Material 1

## Data Availability

The datasets used and/or analysed during the current study are available from the corresponding author on reasonable request.
