# Supplementary material for: Heterogeneity of weight gain after initiation of Elexacaftor/Tezacaftor/Ivacaftor in people with cystic fibrosis
Source: Respir Res. 2023 Jun 17;24:164. doi: 10.1186/s12931-023-02451-0 (PMC10276411; doi:10.1186/s12931-023-02451-0)
Supplement: Supplementary file 1 — Supplementary Material 1 [file 12931_2023_2451_MOESM1_ESM.docx]

Table S1. Demographic and clinical characteristics according to changes in body weight following six months of treatment with elexacaftor/tezacaftor/ivacaftor therapy in patients with cystic fibrosis

| Characteristic | Increase in  BW, N = 79^1^ | No change or decrease in BW, N = 13^1^ | *p*-value^2^ |
| --- | --- | --- | --- |
| Sex |  |  | 0.364 |
| Males | 53 (67.1%) | 7 (53.8%) |  |
| Females | 26 (32.9%) | 6 (46.2%) |  |
| Age (years) |  |  | 0.101 |
| Median (IQR) | 38 (32; 43) | 43 (35; 48) |  |
| Age category |  |  | 0.132 |
| <40 years | 48 (60.8%) | 5 (38.5%) |  |
| ≥40 years | 31 (39.2%) | 8 (61.5%) |  |
| Residual function mutation | 10 (12.7%) | 2 (15.4%) | 0.676 |
| Pancreatic insufficiency | 55 (69.6%) | 9 (69.2%) | >0.999 |
| P. aeruginosa colonization | 49 (62.0%) | 10 (76.9%) | 0.365 |
| Previous CFTR modulator therapy |  |  | 0.041 |
| None | 63 (79.7%) | 6 (46.2%) |  |
| Lumacaftor/Ivacaftor | 14 (17.7%) | 7 (53.8%) |  |
| Tezacaftor/Ivacaftor | 1 (1.3%) | 0 (0.0%) |  |
| Ivacaftor | 1 (1.3%) | 0 (0.0%) |  |
| CFRD | 19 (24.1%) | 4 (30.8%) | 0.730 |
| Height (cm) |  |  | 0.978 |
| Median (IQR) | 170 (163; 174) | 172 (161; 175) |  |
| Weight (kg) |  |  | 0.062 |
| Median (IQR) | 59.0 (54.1; 69.0) | 63.1 (58.5; 72.0) |  |
| BMI (kg/m^2^) |  |  | 0.007 |
| Median (IQR) | 20.9 (19.1; 23.4) | 23.4 (21.8; 25.3) |  |
| BMI category |  |  | 0.041 |
| Underweight | 10 (12.7%) | 0 (0.0%) |  |
| Normal weight | 63 (79.7%) | 9 (69.2%) |  |
| Overweight | 6 (7.6%) | 4 (30.8%) |  |
| ^1^n (%) unless otherwise specified | | | |
| ^2^Fisher's exact test; Wilcoxon rank sum test; Pearson's Chi-squared test  BMI: body mass index. BW: body weight. CFRD: cystic fibrosis-related diabetes. CFTR: cystic-fibrosis transmembrane conductance regulator. IQR: Interquartile range. ppFEV1: percent predicted forced expiratory volume in one second. | | | |
